# Roles of Metabolomics in Allergic Rhinitis: From Cell to Bedside Investigations

**DOI:** 10.3390/ijms27115064

**Published:** 2026-06-03

**Authors:** Pongsathorn Saligupta, Mongkol Lao-Araya, Siriporn C. Chattipakorn, Nipon Chattipakorn, Chanisa Thonusin

**Affiliations:** 1 Division of Allergy and Immunology, Department of Pediatrics, Faculty of Medicine, Chiang Mai University, Chiang Mai 50200, Thailand; pongsathorn.s@cmu.ac.th (P.S.); laoaraya@gmail.com (M.L.-A.); 2 Cardiac Electrophysiology Research and Training Center, Faculty of Medicine, Chiang Mai University, Chiang Mai 50200, Thailand; siriporn.c@cmu.ac.th (S.C.C.); nipon.chat@cmu.ac.th (N.C.); 3 Center of Excellence in Cardiac Electrophysiology Research, Chiang Mai University, Chiang Mai 50200, Thailand; 4 Department of Oral Biology and Diagnostic Sciences, Faculty of Dentistry, Chiang Mai University, Chiang Mai 50200, Thailand; 5 Cardiac Electrophysiology Unit, Department of Physiology, Faculty of Medicine, Chiang Mai University, Chiang Mai 50200, Thailand; 6 The Academy of Science, The Royal Society of Thailand, Bangkok 10300, Thailand

**Keywords:** allergen immunotherapy, allergic rhinitis, allergy, metabolites, metabolomics

## Abstract

Alterations in various metabolic pathways observed in patients with allergies suggest that metabolomics offer a precise and comprehensive approach for the diagnosis of allergic diseases and the monitoring of the efficacy of allergen immunotherapy. The purpose of this review is to provide a comprehensive assessment of the existing evidence regarding metabolomic changes in allergic rhinitis and allergen immunotherapy. The PubMed database search was conducted from inception to December 2025. A narrative synthesis was performed. A total of 16 studies were included. Several metabolic pathways are affected in allergic rhinitis, including amino acid metabolism, fatty acid oxidation, and phospholipid metabolism. Additionally, fatty acids, diacylglycerols, and lysophosphatidylcholines have emerged as potential diagnostic and severity biomarkers for allergic rhinitis. Allergen immunotherapy has also been shown to modulate these metabolic disturbances. Notably, arachidonic acid, linolenic acid, and sphingosine may serve as indicators of therapeutic efficacy. In conclusion, AR is characterized by distinct metabolic alterations and mainly associated with alterations in lipid metabolism. AIT has been shown to modulate metabolic disturbances in AR. Future research should focus on integrating metabolomics with clinical and other molecular approaches to enhance clinical applicability in AR.

## 1. Introduction

Allergic rhinitis (AR) is a common atopic disorder characterized by chronic inflammation of the nasal mucosa due to an exaggerated immune response to environmental allergens [1]. Clinically, AR presents with various symptoms including sneezing, nasal congestion, clear rhinorrhea, and nasal pruritus [2].

Allergen immunotherapy (AIT) is a recommended treatment option for patients with moderate to severe AR [3]. AIT involves the administration of specific allergens via sublingual or subcutaneous routes, with the goal of modulating the immune system to prevent type 2 inflammatory responses being triggered by allergen exposure [4]. AIT inhibit activation and degranulation of mast cells and basophils, resulting in improvement in clinical symptoms, reduced medication use, and sustained therapeutic effects even after treatment cessation [4,5].

Metabolomics is an emerging field within systems biology that focuses on the comprehensive analysis of groups of metabolites, or metabolomes, within biological samples [6]. However, the metabolic alterations underlying AR and those occurring in response to AIT remain incompletely understood.

This review aims to provide an overview of the current applications of metabolomics in AR, with a focus on its potential utility in diagnosis, severity assessment, and monitoring responses to AIT. By integrating metabolomics data with clinical phenotypes and other molecular modalities, we aim to highlight the potential of a metabolomics-based approach in improving the clinical management of AR.

Identified studies were categorized into four key areas, including the following: (1) metabolome alterations in AR, (2) metabolomes and metabolic pathways as diagnostic and severity markers for AR, (3) metabolome alterations following AIT in AR, and (4) metabolomes and metabolic pathways as AIT response markers for AR.

A literature search of the PubMed database was conducted from July 2024 to December 2025 to identify studies published within the previous 10 years. The search strategy included the following terms: “allergen immunotherapy”, “allergic rhinitis”, “allergy”, “metabolites”, “metabolome”, and “metabolomics”.

Studies were included if they (1) were published in English, (2) were published between 2015 and 2025, and (3) reported metabolomic changes in patients with AR or individuals receiving AIT. Studies were excluded if they were systematic reviews, narrative reviews, review articles, opinion pieces, summaries, studies unrelated to AR metabolomics, or studies lacking original metabolomic data.

**Figure 1:**
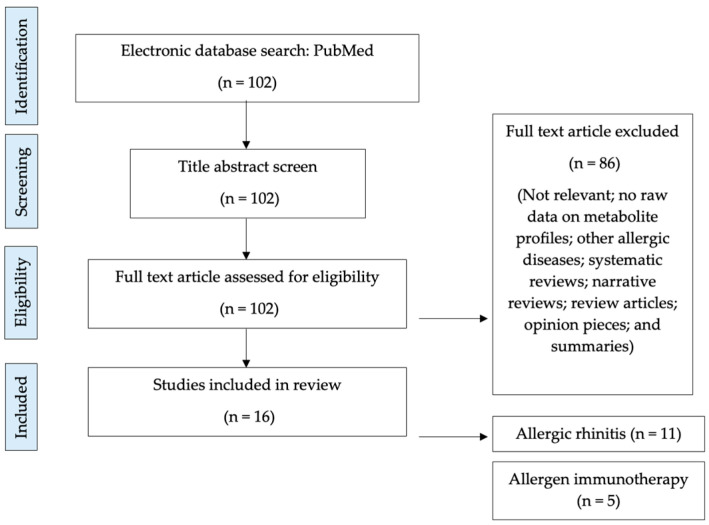
Narrative review flow diagram.

Studies were further evaluated according to methodological clarity, adequacy of metabolomic analysis, and relevance to the objectives of this review. Discrepancies were resolved through discussion and consensus. A total of 102 records were identified, of which 16 studies met the inclusion criteria and were included in the narrative synthesis. A flow chart summarizing the study selection process and reasons for exclusion was added to the manuscript (Figure 1).

## 2. Alterations in Metabolome in Allergic Rhinitis

In the context of AR, eleven metabolomics studies reported significant changes in metabolome profiles. Among these, one was an in vitro study [7], two were in vivo studies [8,9] while the remaining eight were clinical studies [10,11,12,13,14,15,16,17].

In AR, sensitization begins following exposure to allergens, which stimulate the airway epithelium to release alarmins such as thymic stromal lymphopoietin (TSLP), interleukin (IL)-25, and IL-33. These cytokines activate type 2 innate lymphoid cells (ILC2s) and promote dendritic cell-mediated allergen presentation, leading to B-cell activation and immunoglobulin E (IgE) production. Subsequently, IgE binds to high-affinity FcεRI receptors on mast cells and basophils. Upon re-exposure to the allergen, cross-linking of IgE–FcεRI complexes triggers the release of inflammatory mediators, including histamine, prostaglandin D2 (PGD2), leukotriene E4 (LTE4), and leukotriene D4 (LTD4) [18].

These immunologic and inflammatory responses are closely associated with metabolomic alterations observed in AR. The generation of prostaglandins and leukotrienes directly reflects activation of arachidonic acid (AA) and other lipid metabolic pathways, resulting in disturbances in phospholipid, fatty acid, and sphingolipid metabolism [19]. In addition, chronic type 2 inflammation and immune-cell activation increase energy consumption, oxidative stress, and protein turnover [20], thereby contributing to alterations in amino acid metabolism, including pathways related to arginine, ornithine, glycine, and branched-chain amino acids. Collectively, these inflammatory and metabolic interactions suggest that AR is not only an airway inflammatory disorder but also a condition associated with systemic metabolic dysregulation. Although metabolomic findings were heterogeneous across studies, several significant and consistent alterations in lipid metabolism, amino acid metabolism, and inflammation-related pathways have been identified and are discussed in the following sections.

**Figure 2:**
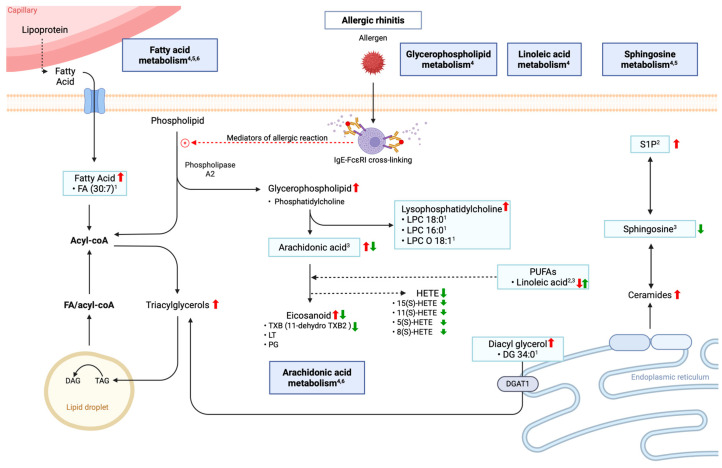
Changes in lipid metabolism in AR and post-AIT; Red arrows represent changes in metabolites in AR patients, whereas green arrows represent metabolite changes post-AIT; ^1^ AUC > 0.9 for AR diagnosis; ^2^ AUC > 0.9 for AR severity; ^3^ AUC > 0.7 for AIT effectiveness; ^4^ Associated pathways for AR diagnosis; ^5^ Associated pathways for AR severity; ^6^ Associated pathways for AIT effectiveness; DAG: Diacylglycerol; FA: Fatty acid; HETE: Hydroxyeicosatetraenoic acid; LPC: Lysophosphatidylcholines; LT: Leukotriene; S1P: Sphingosine-1-phosphate; PG: Prostaglandin; PUFA: Polyunsaturated Fatty Acid; TAG: Triacylglycerol; TXB2: Thromboxane B2. Created with BioRender.com.

**Figure 3:**
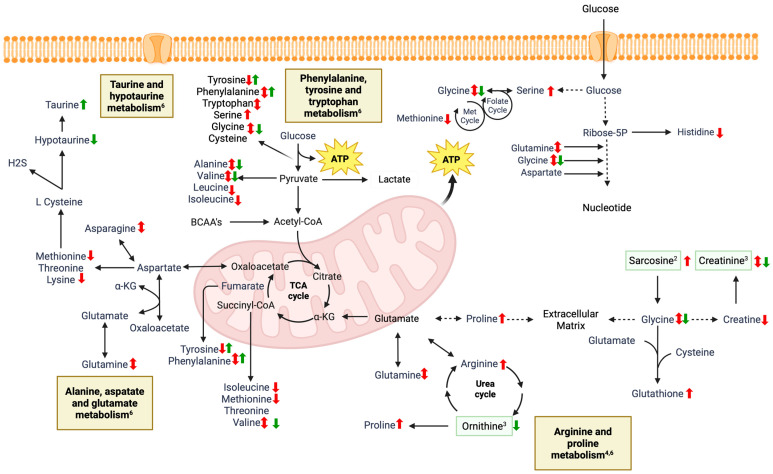
Changes in amino acid metabolism in AR and post-AIT; Red arrows represent metabolite changes in AR patients, whereas green arrows represent metabolite changes post-AIT; ^2^ AUC > 0.9 for AR severity; ^3^ AUC > 0.7 for AIT effectiveness; ^4^ Associated pathways for AR diagnosis; ^6^ Associated pathways for AIT effectiveness; H2S: Hydrogen Sulfide; TCA: tricarboxylic acid. Created with BioRender.com.

These alterations are comprehensively summarized in Table 1 and Table 2 and Figure 2 and Figure 3. Lipid metabolism and amino acid metabolism have been identified as the main systems involved in AR.

### 2.1. Alterations in Lipid Metabolism

- Upregulation of arachidonic acid metabolism

Pathway analysis from humans identified AA and glycerophospholipid metabolism as pathways which were significantly altered in AR [11,13,14]. AA is stored as glycerophospholipids in cell membranes [21]. Upon stimulation (e.g., by cytokines or allergens), phospholipase A2 (PLA2) releases AA from membrane phospholipids [22]. Eicosanoids, which are derived from AA, play pivotal roles in inflammatory responses [23]. These proinflammatory lipid mediators are released from membrane phospholipids through AA metabolism [24]. Previous studies also reported elevated levels of glycerophospholipids and AA-derived eicosanoids—prostaglandins—in the serum of AR patients [13]. Alterations in several leukotrienes and thromboxanes, which are also AA-derived eicosanoids, were also observed in the serum of humans [13,14]. All of these findings suggest that the upregulation of AA metabolism plays a critical role in the promotion of inflammatory responses in AR.

- Upregulation of phosphatidylcholine metabolism

Phosphatidylcholine (PC) and phosphatidylethanolamine (PE) are major membrane phospholipids whose metabolism generates bioactive intermediates such as lysophosphatidylcholine (LPC), lysophosphatidylethanolamine (LPE), and diacylglycerol (DAG), which play key roles in membrane remodeling, inflammation, and intracellular signaling [25]. Increases in LPEs and LPCs have been reported in the serum of a mice model of AR [9]. The capacity of LPE to activate mast cells, macrophages, and airway epithelial cells has been demonstrated [26], while the generation of 12-hydroxyeicosatetraenoic acid- phosphatidylethanolamines (12-HETE-PEs) has been observed in a T helper 2-dependent model of murine lung inflammation, in association with elevated IL-4 and IL-13 levels [27]. From clinical studies, patients with AR showed significantly elevated serum levels of PCs, LPCs, specifically LPC 18:0, LPC 16:0, and LPC(O) 18:1, DAGs species such as DAG 34:0, and PEs [11]. PCs, being among the most abundant phospholipids in membranes, likely reflect ongoing inflammatory processes when their levels are altered [28]. LPCs are known to modulate the effector functions of eosinophils [29]. They promote inflammation by enhancing the production of IL-33 (IL-33) [30,31]. One study demonstrated that LPCs induced key features of allergic airway diseases [30].

Additionally, associations between LPCs, DAGs, triacylglycerols (TGs) and FAs and the immunological markers IgE and IL-33 were revealed in the serum of AR patients [11]. The DAG signaling pathway has been shown to mitigate immune cell and smooth muscle cell dysfunction in asthma, with diacylglycerol kinase (DGK) enzymes contributing to the regulation of airway inflammation and hyperresponsiveness [32]. All of these findings suggest that the upregulation of PC, LPC, LPE and DAG metabolism exerts a key role in promoting inflammatory and allergic responses in AR.

- Increased ceramide and sphingosine-1-phosphate levels

Elevated ceramide levels have been observed in murine mast cells and an AR mice model [7,9], while increased levels of sphingosine-1-phosphate (S1P) have been reported in AR patients [10]. Sphingomyelin (SM) is a major membrane sphingolipid. Upon inflammatory stimulation, acid sphingomyelinase is activated and then catalyzes the hydrolysis of SM into ceramides and phosphatidylcholines [33]. Ceramides are subclasses of phospholipids that play important roles in inflammatory processes [31]. They can be further metabolized by ceramidases into sphingosine, which is subsequently phosphorylated by sphingosine kinases to form S1P [34]. Consistent with findings in AR, ceramides have been shown to accumulate in the lungs and contribute to the development of asthma [35]. Similarly, S1P has been implicated in the pathogenesis of asthma [36]. Given these findings, ceramides and S1P appear to be closely associated with AR as a consequence of allergic inflammation.

### 2.2. Alterations in Amino Acid Metabolism

- Increased arginine and proline levels

Elevated levels of arginine and proline were reported in the serum of an AR murine mast cell model [7]. Pathway analysis in the serum of humans was also linked to AR with dysregulation of arginine and proline metabolism [10]. Arginine, a semi-essential amino acid, plays a critical role in the urea cycle and serves as a precursor for several biologically active molecules, including nitric oxide (NO), creatine, polyamines, and urea [37,38]. Among these, NO is particularly relevant in the context of allergic disease, as it is a well-established biomarker of type 2 inflammation [39]. All of these findings suggest that increased arginine and proline levels in AR reflect heightened NO production and altered nitrogen handling, contributing to inflammatory signaling and immune dysregulation in the nasal mucosa.

- Increased sarcosine and serine, but decreased glycine and creatine levels

Increased sarcosine [10], increased serine [7], and decreased creatine [12,13] were reported in the serum of AR patients. Additionally, three studies reported reduced glycine levels in AR patients, two from serum [12,13] and one from urine [17]. Glycine is recognized for its anti-inflammatory property in various disease models [40] and has been shown to inhibit acute allergic responses in mice [41]. The observed reductions in glycine levels suggested a depletion of anti-inflammatory capacity. Sarcosine, an intermediate metabolites in glycine and creatine biosynthesis, is metabolized into glycine via sarcosine dehydrogenase [42]. Serine is a non-essential amino acid that plays crucial roles in many biological processes [43]. Hence, elevated sarcosine and serine levels, alongside decreased glycine and creatine, suggest an impairment of metabolic conversion and a shift away from the glycine-mediated anti-inflammatory pathway in AR. Moreover, both glycine and serine are involved in glutathione synthesis, which is a potent antioxidant system [44]. In summary, altered levels of these amino acids are likely to be associated with AR via increased oxidative stress and inflammation.

### 2.3. Alterations in Nucleic Acid Metabolism

AR has been reported as being associated with pyrimidine [10] and purine [14] metabolism following pathway analysis. Cytidine, a metabolite involved in pyrimidine metabolism, was found to be elevated in the serum of AR patients [10]. Cytidine serves as a substrate for conversion into uridine via the enzyme cytidine deaminase [45]. Interestingly, this enzyme has been implicated in the pathophysiology of allergic conditions such as food allergy and asthma [46,47]. With regard to purine metabolism, elevated levels of hypoxanthine and uric acid were also observed in the serum of patients with AR [14]. While hypoxanthine is not commonly linked to allergic responses, higher levels of uric acid have been observed in asthmatic patients during acute exacerbation, when compared to asthmatic patients in remission and healthy individuals [48]. For these reasons, the changes in nucleic acid levels in AR are likely to be involved in allergic inflammatory pathways. Further research is needed to identify the relationships between nucleic acid metabolism, inflammation, and AR.

## 3. Metabolomes and Metabolic Pathways as Diagnostic and Severity Markers for Allergic Rhinitis

The diagnosis of AR is traditionally based on clinical symptoms. AR is often confirmed by the identification of sensitization to aeroallergens [49]. Even though additional diagnostic tools such as nasal cytology and nasal allergen challenge tests have been utilized in research, their applications in routine clinical practice remain limited due to their complexity and time requirement [50,51]. AR severity can be assessed through clinical evaluation and quality of life (QOL) using survey instruments and questionnaires, including the Visual Analog Scale (VAS) and the Rhinoconjunctivitis Quality of Life Questionnaire (RQLQ) [52]. Currently, there is no validated laboratory marker for the assessment of AR severity. For these reasons, metabolomics offers a promising and minimally invasive approach to support the diagnosis of AR and severity assessment, particularly if validated biomarkers are established.

Pathway analysis, correlation analysis, and receiver operating characteristic (ROC) analysis are powerful tools for the identification of potential metabolic biomarkers [53]. Integration of these analytical approaches can reveal key metabolites among candidate biomarkers in AR. To date, four studies have reported candidate metabolites with potential diagnostic value in AR [10,11,13,14]. Two of these also identified metabolites associated with disease severity [10,11]. A summary of the findings is provided in Table 3.

### 3.1. Lipid Metabolites

For the diagnosis of AR, pathway analysis has consistently identified four key lipid-related metabolic pathways, including (1) AA metabolism [10,11,13,14], (2) glycerophospholipid metabolism [10,11], (3) linoleic acid metabolism [11,13], and (4) sphingolipid metabolism [10]. Furthermore, one of these studies discovered five lipids with excellent diagnostic performance (area under the curve; AUC > 0.9), including increased fatty acid (FA) 30:7, LPC(O) 18:1, DAG 34:0, LPC 18:0, and LPC 16:0 [11].

For severity assessment, lipids again showed potential as candidate biomarkers. Specifically, increased FA, DAG, LPC, and TG levels showed a correlation with clinical severity, as indicated by increased serum IgE and IL-33 levels [11].

In AR, lipid metabolism plays a central role in driving inflammation, as previously mentioned. Together, the increases in these lipid molecules reflect the inflammatory state of AR and may serve as potential biomarkers for disease diagnosis and severity.

Another study [10] revealed an increase in S1P and a decrease in linoleic acid potentially served as severity-associated markers, as indicated by total nasal symptom score (TNSS) and VAS score with both achieving an AUC greater than 0.9. Linolenic acid, a common polyunsaturated fatty acid, plays an important role in the modulation of the immune responses in allergic and inflammatory diseases [54]. A previous study showed that the linolenic acid exerted immunosuppressive effects by reducing mast cell activation and secretion [55]. Consequently, linolenic acid might help in the prevention and treatment of various inflammatory conditions. S1P is a bioactive lipid molecule derived from sphingolipid metabolism and is involved in inflammatory processes [56]. S1P is also involved in the development of the asthma phenotype in patients allergic to house dust mites (HDM) [57]. Therefore, a decrease in linolenic acid and an increase in S1P levels may serve as potential biomarkers for the severity of AR via the modulation of inflammatory processes.

### 3.2. Other Metabolites

In addition to lipids, arginine and proline metabolism [10], purine [14] and pyrimidine [10] metabolism, as well as in caffeine and porphyrin metabolism [13,14] exerted diagnostic potential for AR according to pathway enrichment analysis.

Arginine and proline metabolism are closely linked through enzymatic pathways [58]. Arginine is essential for the urea cycle and acts as a precursor for NO [37,38], a key biomarker of type 2 inflammation associated with allergic disease [39]. Purine and pyrimidine nucleotides serve as key immune signaling molecules [59], and alterations in purine metabolism observed in asthma indicate enhanced purine turnover linked to chronic inflammation [60]. Caffeine, a structural analog of adenosine, functions as a non-selective adenosine receptor antagonist and is also linked to the development of asthma [61]. Interestingly, higher levels of paraxanthine, a primary metabolite of caffeine, in urine are associated with reduced asthma risk and improved pulmonary function in non-asthmatic adults, whereas elevated urine levels of caffeine and its metabolite theophylline are linked to unfavorable pulmonary function in those with asthma [62]. These findings highlight the potential of metabolites involved in purine, pyrimidine, and caffeine metabolism in diagnosing allergic diseases.

With regard to severity assessment, one study [10] revealed that increased sarcosine and cytidine potentially served as severity-associated markers, as indicated by TNSS and VAS scores. Sarcosine, a glycine derivative, is also associated with asthma, as shown by observed elevated serum sarcosine levels in asthma patients [63]. Cytidine, a pyrimidine precursor of cytidine triphosphate (CTP), is essential for nucleic acid and lipid metabolism, and dysregulation of pyrimidine metabolism has also been linked to asthma pathophysiology [64]. All of these results emphasized the roles of sarcosine and cytidine in determining the severity of allergic diseases.

Although all these findings are promising, further validation in large and well-characterized clinical cohorts are necessary to establish the utility of metabolomics as a tool for the routine diagnosis and severity assessment of AR.

## 4. Metabolome Alterations Following Allergen Immunotherapy in Allergic Rhinitis

In AIT, repeated administration of high doses of allergens through sublingual immunotherapy (SLIT) or subcutaneous immunotherapy (SCIT) modulates the immune response toward immune tolerance. During this process, dendritic cells induce naïve CD4+ T cells to differentiate into T-helper 1 (Th1) cells and regulatory T cell subsets, including Treg, Tr1, and Tr35 cells. Regulatory T cells subsequently produce inhibitory cytokines and immune-regulatory molecules, such as cytotoxic T-lymphocyte-associated protein 4 (CTLA-4), IL-10, IL-35 and transforming growth factor-β (TGF-β), which suppress T-helper 2 (Th2)-mediated allergic inflammation.

In addition, IL-10 and TGF-β promote B-cell differentiation into regulatory B cells (Bregs) and plasma cells, leading to increased production of allergen-specific immunoglobulin G (IgG), IgG4, and immunoglobulin A (IgA). Bregs further suppress IgE production by B cells. Allergen-specific IgG/IgG4 and IgA compete with IgE for allergen binding, thereby reducing mast cell and basophil activation and degranulation [4]. Overall, these immunologic mechanisms suppress the pathophysiology of AR.

Therefore, successful AIT may reverse or normalize metabolomic alterations associated with AR. However, the therapeutic response to AIT may vary depending on multiple factors, including host characteristics, route of allergen administration, allergen dose, allergen type, and duration of immunotherapy. The differences between SCIT and SLIT include a greater reduction in IgE levels and a more pronounced increase in IgG4 levels with SCIT compared with SLIT [65]. The metabolomic changes associated with AIT are discussed further in the following sections.

Five clinical studies reported a range of metabolites that were altered following AIT [66,67,68,69,70]. Grass pollens were used as allergens in three studies, Artemisia sieversiana for SCIT in two studies and Phleum pratense for SLIT in one study. HDM were also used as allergens in two studies, including one SLIT and one SCIT. The maintenance phase of AIT ranged from one to three years. All metabolome alterations following AIT in AR are comprehensively summarized in Table 4. Of these, lipid metabolism, amino acid metabolism, and glycolysis metabolism are the main processes involved.

### 4.1. Alterations in Lipid Metabolism

In three studies, following AIT, a reduction in serum AA level was reported [67,68,69], while decreases in serum HETEs, downstream metabolites of AA, were observed in one study [67]. Levels of AA and other polyunsaturated fatty acids (PUFAs), precursors for eicosanoids involved in AR symptomatology [71], were also significantly reduced in serum after AIT [67,68,69]. For these reasons, the decreases in AA and HETEs are likely to indicate a corresponding decrease in eicosanoid production. Pathway analysis also revealed that the AA metabolism pathway underwent significant modulation post-AIT [69].

Another study reported that serum sphingosine level was significantly lower in an AIT-effective group than that of an ineffective group following treatment [68]. Sphingosine is a bioactive lipid involved in sphingolipid metabolism that has been implicated in allergic inflammation. It has also been reported as being a relevant metabolite in other allergic conditions such as atopic dermatitis [72] and asthma [57]. All of these findings suggest that the lower level of sphingosine in the AIT-effective group is associated with the attenuation of allergic inflammation. However, further studies evaluating the correlations between sphingosine and inflammatory markers in AR patients treated with AIT is required.

### 4.2. Alterations in Amino Acid Metabolism

- Decreased ornithine and creatinine levels

Reductions in serum ornithine and creatinine levels following AIT were observed [68]. These metabolites are downstream products of arginine metabolism [73], which plays a central role in the urea cycle and is closely linked to NO production [74]. NO serves as a key mediator of eosinophilic inflammation and is recognized as a biomarker of nasal inflammation in AR patients [75]. Hence, the decline in ornithine and creatinine levels post-AIT possibly reflects a downregulation of arginine metabolism and a corresponding decrease in NO production, suggesting a reduction in allergic inflammatory activity. This statement is supported by a previous study which showed that elevated levels of ornithine and creatinine were associated with exacerbation of asthma [63].

- Increased taurine and decreased hypotaurine levels

Following AIT, an increase in the serum level of taurine and a decrease in serum hypotaurine was found in one study [66]. Taurine and hypotaurine are key metabolites in the sulfur-containing amino acid metabolic pathway. Hypotaurine is converted into taurine through the enzymatic activity of hypotaurine dehydrogenase [76]. Taurine is known to possess potent antioxidant, anti-inflammatory, and anti-apoptotic properties [77]. In a human mast cell model, taurine suppressed mRNA expression of TSLP and pro-inflammatory cytokines in a dose-dependent manner [78]. The observed metabolic shift, characterized by a decrease in hypotaurine and a corresponding increase in taurine, suggests an anti-inflammatory effect of AIT in AR patients. This alteration in sulfur amino acid metabolism has been supported by both pathway analysis and univariate statistical approaches [66].

### 4.3. Alterations in Glycolysis

A decrease in serum lactate level following AIT was exhibited in one study [68]. Lactate, a byproduct of glycolysis and pyruvate metabolism [79], is usually elevated in response to tissue hypoxia and inflammation [80]. Therefore, the reduction in lactate level following AIT is likely to reflect a decrease in inflammatory activity.

## 5. Metabolomes and Metabolic Pathways as Allergen Immunotherapy Response Markers for Allergic Rhinitis

The clinical effectiveness of AIT in AR patients is typically evaluated following a one-year treatment through symptom-based measures such as the TNSS, the RQLQ, and the VAS. A meta-analysis has revealed that significant clinical improvement or a reduction in medication use are surrogate markers of AIT response [81]. In cases where no clinical improvement is observed, the patient is considered unresponsive to AIT, and thus discontinuation of therapy is recommended [82]. Conversely, if a positive clinical response is noted, AIT is continued for a duration of 3 to 5 years [82]. Unfortunately, standardized laboratory-based methods for assessing AIT effectiveness remain limited. A blood-based metabolomics profile may offer a more objective and simplified approach for the monitoring of treatment response.

To identify candidate biomarkers associated with AIT responsiveness, pathway analysis, correlation analysis, univariate analysis, and ROC analysis have been performed in patients with AR [53]. To date, five studies have identified metabolites which are potential candidates associated with AIT efficacy [66,67,68,69,70]. A summary of these findings is presented in Table 5.

### 5.1. Lipid Metabolism

Pathway analysis showed that two lipid-related metabolic pathways, AA metabolism [69] and fatty acid metabolism [68], were associated with the therapeutic response to AIT. Since these pathways have also been implicated in AR diagnosis and severity, it is highly suggestive that AIT reverses the metabolic dysregulation characteristics of AR. Correlation analyses enabled the identification of several metabolites significantly associated with good response to AIT, including decreased 11-dehydrothromboxane B2 (11-dehydroTXB2), 11(S)-HETE, 5(S)-HETE, 8(S)-HETE, 15(S)-HETE, and 13-hydroxyoctadecadienoic acid (13-HODE). It has been shown that 11-dehydroTXB2, a stable metabolite of thromboxane A2 [83], HETEs, metabolites of AA [84] and 13-HODE, a lipid metabolite derived from linoleic acid [85], are associated with the inflammatory process. One study [68] utilized ROC analysis to evaluate the predictive value of metabolites for the efficacy of AIT. They reported that a decreased level of AA and sphingosine, along with increased linoleic acid, demonstrated predictive potential (AUC > 0.7). Eicosanoids derived from AA are central mediators of inflammation, generated from membrane phospholipids through AA metabolism [24]. Sphingosine itself is a component of cell membranes, but its derivative, S1P, is a critical lipid mediator involved in inflammation [56]. Therefore, it is likely that the decreases in these metabolites represent a good therapeutic response to AIT via the reduction in allergic inflammation.

### 5.2. Amino Acid Metabolism

Amino acids have also emerged as important biomarkers in predicting the efficacy of AIT. These amino acids included alanine, aspartate, and glutamate, as identified through pathway analysis. Moreover, pathway analysis has been used to demonstrate that aminoacyl-tRNA biosynthesis, arginine and proline metabolism, butanoate metabolism, nitrogen metabolism, phenylalanine, tyrosine and tryptophan biosynthesis, and also taurine and hypotaurine metabolism could reflect AIT responsiveness [70]. In addition, univariate analysis has specifically highlighted that a decreased level of hypotaurine and l-alanine, along with increased taurine indicate a good response to AIT [66,68,70].

Hypotaurine is converted to taurine by hypotaurine dehydrogenase, and taurine is subsequently metabolized to L-alanine [76]. Taurine exerts antioxidant, anti-inflammatory, and anti-apoptotic effects [77]. Therefore, the shift from hypotaurine to taurine after AIT may be mediated by these beneficial effects.

One study [68] used ROC analysis and found that a decrease in creatinine and ornithine was associated with AIT efficacy, demonstrating predictive utility (AUC > 0.7). Creatinine, a breakdown product of creatine phosphate, can reflect cellular energy metabolism and systemic inflammation [86]. Ornithine is a non-protein amino acid produced from arginine via arginase in the urea cycle [87]. In allergic inflammation, levels of L-ornithine are increased [88]. For these reasons, the reduction in creatinine and L-ornithine may be associated with a decrease in allergic inflammation, and hence these changes can serve as potential markers of AIT efficacy.

### 5.3. Other Metabolic Pathways

In addition to lipids and amino acids, other metabolic pathways are also shown to be AIT response markers as evidenced by pathway analysis. This included glycolysis, pyruvate metabolism, galactose metabolism, TCA cycle, and the pentose phosphate pathway [66,68,69,70]. ROC analysis was used to evidence that decreased lactate level was associated with a good response to AIT [68]. Lactate, produced from pyruvate during anaerobic glycolysis [89], is elevated in asthma [90]. Acting as an immune modulator, lactate can promote the differentiation of naive CD4^+^ T cells into inflammatory T-helper 17 (Th17) cells [91]. AIT may normalize lactate levels, suggesting its potential as a marker of a positive therapeutic response via the attenuation of inflammation.

## 6. Limitations and Future Direction

Although metabolomics techniques have advanced rapidly, clinical applications in AR and AIT remain in early stages and are largely confined to research settings. No single metabolite can demonstrate sufficient diagnostic, prognostic, and therapeutic monitoring utility across all aspects of AR, which include disease presence, severity, and responsiveness to AIT. Therefore, combining conventional biomarkers (e.g., skin prick tests, serum-specific IgE) with metabolomics profiling is likely to offer a more comprehensive and accurate approach. In addition, a major challenge in this field is the lack of standardization in metabolomics methodologies, which impairs reproducibility and limits the comparability of findings across studies. The inherent complexity and heterogeneity of AR phenotypes and AIT protocols also contribute to significant variability in study populations.

In an in vitro study [7], LPS-sensitized murine mast cells were investigated, whereas in vivo studies [8,9] used ovalbumin (OVA)-sensitized AR mouse models. These studies reported metabolite alterations following sensitization, including increases in fatty acids and lipid metabolites. In addition, pathway analysis demonstrated increased AA and sphingolipid metabolism in the murine mast cell model [7]. These findings were similar to those observed in human studies, as discussed in the following section. However, several limitations of non-clinical studies should be considered. First, the experimental models were sensitized with allergens such as OVA, whereas human AR is more commonly associated with sensitization to HDM [92]. Second, these studies lacked clinical correlations between metabolite alterations and AR severity. Finally, the number of non-clinical metabolomics studies in AR remains limited.

Samples used for metabolomic analyses in in vivo studies included serum and feces, whereas clinical studies analyzed serum, sputum, and urine samples. One study [17] reported that stool metabolites were strongly positively correlated with blood metabolites, while blood metabolites were negatively correlated with most urine metabolites. This may be explained by the absorption of dietary components into the bloodstream, whereas unabsorbed compounds and metabolic byproducts are excreted in the stool. In addition, the liver processes numerous metabolites and excretes them into bile before entering the gastrointestinal tract, resulting in parallel metabolic representations in both blood and fecal samples. In contrast, the kidneys regulate metabolite homeostasis by adjusting urinary excretion, which may lead to reduced metabolite concentrations in urine samples. Therefore, the interpretation of metabolite alterations should be performed cautiously, with consideration of the specific biological specimen analyzed.

Different metabolomic platforms, including liquid chromatography coupled with mass spectrometry (LC/MS), gas chromatography coupled with mass spectrometry (GC/MS), and nuclear magnetic resonance spectroscopy (NMR), have distinct analytical characteristics that may contribute to variability among studies. LC/MS provides high sensitivity and broad metabolite coverage [93], particularly for lipids and polar metabolites, whereas GC/MS is more suitable for volatile and thermally stable compounds, such as organic acids and amino acids, and often requires chemical derivatization before analysis [94]. In contrast, NMR offers high reproducibility and minimal sample preparation but has lower sensitivity compared with mass spectrometry-based techniques [95].

Additionally, studies can use either targeted or untargeted metabolomics. Targeted metabolomics focuses on the quantitative analysis of predefined metabolites with high specificity and sensitivity, while untargeted metabolomics aims to comprehensively identify a wide range of metabolites without prior selection, enabling the discovery of novel metabolic alterations [96]. These methodological differences may influence metabolite detection, quantification, and pathway interpretation across studies, and therefore should be considered when comparing metabolomic findings between studies in AR.

In clinical studies, there were differences in the study populations. For example, two studies were conducted in children [11,17], whereas the others included adult participants. Children usually exhibit more Th2-skewed immune responses [97], which are associated with IgE production and eosinophilic inflammation. Moreover, differences were also observed in sensitization patterns and the choice of allergens used for AIT, such as HDM and pollens. These variations may influence metabolomic profiles and treatment responses across studies.

Since AR is usually diagnosed clinically, simpler and more accessible tools, for example, allergen-specific IgE and skin tests, are typically preferred in routine clinical practice. Moreover, blood, urine, and fecal metabolomic profiles reflect systemic metabolic changes, making them susceptible to confounding by comorbid conditions such as infections, inflammation, or other allergic diseases. All of these aspects underscore the need for careful interpretation and clinical contextualization of metabolomics data.

Future research should aim to address these limitations through well-designed clinical and real-world studies. This includes efforts to correlate metabolome profiles with clinical assessments, both subjective and objective, in addition to immunologic markers and treatment outcomes. Additionally, studies should evaluate the metabolic effects of pharmacotherapies used in AR. In other words, the application of pharmacometabolomics to identify appropriate therapeutic agents and to predict patient responsiveness represents a promising approach which is in alignment with the principles of precision medicine. Comparative analyses of metabolomics signatures across different allergen sensitizations, such as house dust mites, animal dander, fungi, and pollen, are also warranted. Further investigations should also examine metabolic differences between AR and other allergic conditions, including asthma, food allergy, drug allergy, and urticaria, as well as in patients with multiple concurrent allergic diseases. Moreover, research should assess the metabolic effects associated with various routes of AIT administration, including sublingual, subcutaneous, and intralymphatic approaches. Robust meta-analyses in addition to both internal and external validation studies are essential to confirm and generalize metabolomics findings for clinical application. Importantly, future efforts should focus on the development of a standardized, clinically applicable metabolomics platform for diagnosis, stratifying severity, and monitoring the effectiveness of AIT in AR. Given the vast and complex nature of metabolomics data, the incorporation of artificial intelligence and machine learning tools are likely to be instrumental in managing, interpreting, and applying these data effectively.

## 7. Conclusions

AR is characterized by distinct metabolic alterations, as summarized in Figure 2 and Figure 3. AR is mainly associated with alterations in lipid metabolism, amino acid metabolism, glycolysis, TCA cycle, and nucleic acid metabolism. These include increases in AA and PC metabolism, elevated ceramide and S1P levels, higher levels of arginine, proline, sarcosine, and serine, and reduced glycine and creatine. Specific metabolites have been identified as potential biomarkers for AR. Indeed, FAs, DAGs, and LPCs are considered diagnostic markers, while S1P, linoleic acid, sarcosine, and cytidine exert potential as severity markers. AIT has been shown to modulate metabolic disturbances in AR, possibly contributing to the alleviation of clinical symptoms. Furthermore, specific metabolites, AA, linolenic acid, sphingosine, creatinine, ornithine, and lactic acid, can be potential biomarkers for assessing the efficacy of AIT in AR.

## Figures and Tables

**Table 1:** Metabolome alterations in allergic rhinitis: Evidence from preclinical studies.

Study Population	Sensitization	Method and Sample	Categories of Metabolites/Metabolic Pathways	Changes in Metabolites		Impacted Pathway from Pathway Analysis	Interpretation	Citation
				Increase	Decrease			
In vitro study								
Murine mast cells	LPS (10 μg/mL for 4 h)	Untargeted and targeted LC/MS in mast cell	Amino acids, peptides, and their related-metabolites	Glutathione Oxiglutatione L-arginine L-asparagine L-glutamine L-phenylalanine L-proline L-serine L-valine	L-histidine	Phenylalanine Tyrosine Tryptophan Histidine Arachidonic acid Sphingolipid Glycine Serine Threonine	AR was associated with increased arachidonic acid and sphingolipid metabolism, whereas histidine metabolism was decreased	[7]
			Fatty acids and fatty acyls	8S,15S-diHPETE 9-HpETE Dinoprost Epoprostenol Arachidonic acid				
			Leukotrienes	Leukotriene A4 Leukotriene D4 Leukotriene E4 Leukotriene B3 Leukotriene B4 Leukotriene B5				
			Thromboxanes	Thromboxane A2 Thromboxane A3 Thromboxane B2				
			Sphingolipids	CerP(d18:1/20:0) Cer(d18:1/16:0) Cer(d18:1/14:0)				
			Glycolysis and sugars	DHAP(18:0)	Aminofructose 6-phosphate			
			Others	Ethyl pyruvate Histamine Retinoic acid	Lipoxin C4			
In vivo study								
AR mice	OVA (40 μg OVA injection on day 0, 7, 14 and 21)	Untargeted LC-MS/MS in serum and feces	Serum				AR was associated with decreased glycolysis and TCA cycle metabolism in serum, but increased amino acid, glycolysis, and TCA cycle metabolism in feces. However, alpha-linolenic acid levels were consistently decreased in both the serum and feces of AR mice.	[8]
			Amino acids, peptides, and their related-metabolites	L-tryptophan	L-alanine	Serum TCA cycle Bile secretion Central carbon Feces TCA cycle		
			Fatty acids and fatty acyls	3-phenylpropanoic acid Myristoleic acid 9,10 DiHOME 2-hydroxy-butanoic acid	Alpha-linolenic acid Arachidonic acid Docosahexaenoic acid 12(R)-HETE			
			Glycolysis and sugars		D-mannose Sedoheptulose D-lyxose			
			Nucleic acids	2 Deoxyuridine Thymidine	Xanthosine			
			TCA cycle		Dihydroxyacetone Pyruvaldehyde Myo-inositol D-allose L-malic acid			
			Bile Acids	Taurochenodeoxycholate Cholic acid Deoxycholic acid				
			Others	Urocanic acid	DL-lactate Phosphorylcholine			
			Feces					
			Amino acids, peptides, and their related-metabolites	L-alanine Dimethylglycine N Acetyl DL-methionine Pantothenic acid				
			Fatty acids and fatty acyls	Isobutyric acid Propionic acid (S)-2-hydroxyglutaric acid Citraconic acid 15 Keto PGE1, 3-phenylpropanoic acid Docosahexaenoic acid 2-oxoadipic acid	Linolenic acid 9R,10S EpOME			
			Glycolysis and sugars	D-mannose D-ribose D-(+)-melibiose Adynerin Glyceric acid Galacturonic acid D-threitol				
			Nucleic acids	Purine Xanthosine Ribothymidine Guanosine	Oxypurinol			
			TCA cycle	Alphaketoglutate Dihydroxyacetone				
			Bile acids		Deoxycholic acid			
			Other organic acids	Acamprosate				
			Others	Homoveratric acid Urocanic acid				
AR mice	OVA (20 μg OVA injection on day 0, 2, 4, 6, 8, 10 and 12)	Untargeted LC/MS in serum	Amino acids, peptides, and their related-metabolites	L-phenylalanine L-arginine Aspartyl-serine	D-tryptophan Homocysteine D-glutamine D-asparagine		AR was associated with phenylalanine, tyrosine and tryptophan, phenylalanine and arachidonic acid metabolism detected in serum.	[9]
			Fatty acids and fatty acyls	Palmitic amide Arachidonic acid				
			Glycolysis and sugars		Acetylglycine			
			Phospholipids	LPE (0:0/20:0) LPC (15:0)				
			Sphingolipids	Cer (d18:0/18:0)				
			Nucleic acids	Uric acid	Uridine Adenine			
			Others	Corticosterone				

8S,15S-diHPETE, 8S,15S-dihydroperoxy-5Z,9E,11Z,13E-eicosatetraenoic acid; 9-HpETE, 9-hydroperoxy-5,7E,11Z,14Z-eicosatetraenoic acid; 9(10)-EpOME, 9,10-epoxy-12-cis-octadecenoic acid; 9R,10S EpOME, (9R,10S)-9,10-Epoxyoctadecenoic acid; AR, allergic rhinitis; C, control; Cer, Ceramide; CerP, Ceramide 1 phosphate; DHAP, dihydroxyacetonephosphate; HETE: hydroxyeicosatetraenoic acid; LC, liquid chromatography; LC/MS: Liquid Chromatography coupled with mass spectrometry; LC/MS/MS: Liquid chromatography coupled with tandem mass spectrometry; LPS, lipopolysaccharide; LPE: Lysophosphatidylethanolamines; LPC: Lysophosphatidylcholine; MS, mass spectrometry; OVA, ovalbumin; PC: Phosphatidylcholine; PE: Phosphatidylethanolamines; PGE, Prostaglandin E; PGE1, Prostaglandin E1; TCA, Tricarboxylic acid cycle.

**Table 2:** Metabolome alterations in allergic rhinitis: Evidence from clinical studies.

Study Population	Sensitization	Method and Sample	Categories of Metabolites/Metabolic Pathways	Changes in Metabolites		Other Findings	Interpretation	Citation
				Increase	Decrease			
C: 28.5 ± 8.5 (29) vs. MAR ^1^: 28.2 ± 9.6 (30) vs. MSAR ^1^: 30:4 ± 8.4 (42)	HDM	Untargeted LC/MS in serum	MAR ^1^ vs. C				AR was associated with the increase in phospholipid and sphingolipid metabolism detected in serum, while levels of sarcosine, S1P, cytidine and linoleic acid detected in serum were associated with its severity.	[10]
			Amino acids, peptides, and their related-metabolites	Sarcosine ^4^ 5′-methylthioadenosine 5-methoxyindoleacetate Creatinine	L-methionine			
			Fatty acids and fatty acyls	Palmitic acid	Trans-vaccenic acid Arachidic acid			
			Phospholipids	Triethanolamine				
			Sphingolipids	S1P ^4^				
			MSAR ^1^ vs. C					
			Amino acids, peptides, and their related-metabolites	2-oxoadipic acid Betaine Sarcosine ^4^ 1,3-diaminopropane				
			Fatty acids and fatty acyls	Cis-9-palmitoleic acid	Linoleic acid ^4^			
			Phospholipids	Phosphorylcholine				
			Sphingolipids	S1P ^4^				
			Nucleic acids	Cytidine ^4^				
			Bile acids		Taurocholic acid			
			MSAR ^1^ vs. MAR ^1^					
			Amino acids, peptides, and their related-metabolites	Betaine Sarcosine ^4^	Pyroglutamic acid			
			Fatty acids and fatty acyls		Linoleic acid ^4^ Palmitoleic Trans-vaccenic acid			
			Sphingolipids	S1P				
			Nucleic acids	Cytidine ^4^				
			Others	D-glucurono-6,3-lactone	Coumarin			
C: 8.77 ± 2.50 (44) vs. PR: 7.84 ± 3.33 (43) vs. NR: 7.91 ± 3.22 (32)	Inhalant allergen	Untargeted LC/MS in serum	AR vs. C				AR and its severity were associated with an increase in lipid metabolism detected in serum.	[11]
			Fatty acids and fatty acyls	FA (20:4), (30:7) ^2^ NAE (22:5)				
			Phospholipids	LPC (18:0) ^2^, (16:0) ^2^, (20:1) LPC(O) (18:2), (18:1) ^2^, (18:0), (16:1), (16:0) PE (36:4), (38:6) LPE (16:0) PS (38:4)				
			Other lipids	TG (56:2), (54:8), (54:4), (52:5), (60:3), (58:3), (58:2), (56:1), (54:7) DAG (38:5), (36:5), (36:3), (36:2), (36:1), (34:0) ^2,3^, (38:6), (36:4), (34:2), (34:1) CL (62:2) ^3^				
			NR vs. PR					
			Other lipids	DAG (34:0), (32:0), (36:0), (42:6) CL (62:6)				
Pollen season vs. Remission	Pollen allergen	Untargeted ^1^H NMR in serum	Amino acids, peptides, and their related-metabolites	N-acetylglutamine	Isoleucine Leucine Valine Allothreonine Alanine Methionine Glutamine Lysine Glycine L-tyrosine Histidine Phenylalanine Creatine Creatinine		AR and its severity were mainly associated with alterations in amino acid and lipid metabolism detected in serum.	[12]
			Fatty acids and fatty acyls		3-hydroxybutyric acid			
			Glycolysis		Lactate			
			Others	Isopropanol	Acetate O-acetyl-choline			
C: 34.8 ± 3.2 (15) vs. AR: 39.8 ± 2.7 (28)	Seasonal allergen	Untargeted LC/MS in serum	Amino acids, peptides, and their related-metabolites		L-tryptophan	Respiratory microbiomes ∝ Serum metabolome levels	AR was associated with alterations in linoleic acid, arachidonic acid, and caffeine metabolism detected in serum, which might be mediated by the alterations in respiratory microbiomes.	[13]
			Linoleic acid metabolism	Linoleic acid	9,10-epoxyoctadecenoic acid 12,13-EpOME			
			Other fatty acids and fatty acyls	Oleic acid Docosahexaenoic acid				
			Glycolysis and sugars		D-glucose			
			Phospholipids		Glycero phosphocholine PA (P-16:0/18:2(9Z,12Z))			
			Arachidonic acid metabolism	Prostaglandin E2 Prostaglandin H2 Prostaglandin D2	Thromboxane A2 20-hydroxy-leukotriene B4			
			Nucleic acids	Deoxyuridine	Inosine			
			Bile acids		Chenodeoxycholic acid Taurochenodesoxycholic acid			
			Caffeine metabolism		Paraxanthine Theobromine			
			Others	Bilirubin 6-thioxanthine 5′- monophosphate	Coproporphyrin Glycine conjugate Pregnenolone sulfate Dehydroepiandrosteronesulfate Presqualene diphosphate			
C: 42.52 ± 6.44 (28) vs. AR: 45.13 ± 7.85 (28)	Aeroallergen	Targeted LC/MS in serum	Amino acids, peptides, and their related-metabolites		N-succinyl-L-diaminopimelic acid		AR was associated with alterations in porphyrin-chlorophyll, arachidonic acid, and purine metabolism detected in serum.	[14]
			Fatty acids and fatty acyls	15(S)-HETE Hexadecanoic acid	13(S)-HPODE			
			Phospholipids		Leukotriene D4			
			Nucleic acids	Hypoxanthine Urate				
			Others	Bilirubin	Stercobilinogen Chlorophyll B			
C: 36.93 ± 3.50 (14) vs AR: 41.21 ± 1.85 (14)	Aeroallergen	Untargeted LC in sputum	Amino acids, peptides, and their related-metabolites	Ergothioneine N-arachidonoyl-l-alanine	Epoxomicin L-tryptophan		AR was mainly associated with alterations in amino acid, fatty acid, lipid, nucleic acid, TCA cycle, and bile acid metabolism detected in sputum.	[15]
			Fatty acids and fatty acyls	1,3-diaminopropane acetol 13,14-dihydro-15-ketoprostaglandin d1				
			Other lipids	Manoalide (-)-perillyl alcohol Lupenone Zerumbone	Hyperforin 10-deacetylbaccatin III			
			Nucleic acids	S-methyl-5′-thioadenosine Thymine Barbituric acid	Inosine			
			TCA cycle	Polygodial	Succinate			
			Other organic acids		Didodecyl 3,3′’-dithiodipropionate Spiculisporic acid			
			Bile acids	Chenodeoxycholic acid				
			Others	Zinniol Echimidine Acebutolol Scutellarein,DI 4-hydroxy-3-methoxymandelic acid Guanidine	3-methylbenzyl alcohol Procyanidin A2 Phenytoin Aminophenazone			
AR: 41.4 ± 10.2 (41)	Ragweed	^1^H NMR in urine	After allergen challenge				AR and its severity were associated with alterations in amino acid, fatty acid, glycolysis, nucleic acid, TCA cycle, and organic acid metabolism detected in urine.	[16]
			Amino acids, peptides, and their related-metabolites	Glycine	Pyroglutamate			
			Fatty acids and fatty acyls	Glycolic acid				
			Glycolysis and sugars	Tartrate	Xylose			
			Nucleic acids		Hypoxanthine			
			TCA cycle		Succinate			
			Other organic acids	Formate Trans-aconitate	1-methylnicotinamide			
C: 4.9 ± 0.8 (24) vs. AR: 4.9 ± 0.5 (26)	Der p and Der f	^1^H NMR in stool, blood and urine	Blood			Stool IgE level ∝ Blood Isovaleric acid	Levels of urine alanine, N,N-dimethylglycine, and chlophedianol, along with levels of blood isovaleric acid, ethanol, and acetylcarnitine were associated with AR.	[17]
			Amino acid and peptides	Phenylalanine				
			Fatty acids and fatty acyls	Isovaleric acid				
			Urine					
			Amino acids, peptides, and their related-metabolites		N,N Dimethylglycine Alanine			
			Others	Chlophedianol				

^1^ MAR and MSAR were categorized according to ARIA criteria; ^2^ ROC curves of the differential lipids (AR vs. C) with an AUC > 0.9; ^3^ NR > C and NR > PR; ^4^ Area under the curve (AUC) value higher than 0.9 for AR severity; 12,13-EpOME, 12,13-cis-Epoxyoctadecenoic acid; 13(S)-HPODE,13(S)-Hydroperoxyoctadecadienoic Acid; ∝, Association; AR, allergic rhinitis; AS, Asthma; AUC, area under the curve; C, control; CL: cardiolipin; DAG: diacglycerol; EPA: eicosapentaenoic acid; FA: fatty acids; HDM, House dust mite; HETE, hydroxyeicosatetraenoic acid; IgE, Immunoglobulin E; IL, Interleukin, LC: Liquid chromatography; LC/MS: Liquid chromatography coupled with mass spectrometry; LPI: Lysophosphatidylinositols; LPC: Lysophosphatidylcholines; LPC(O): ether-linked lysophosphatidylcholine; LPE: Lysophosphatidylethanolamines; MAR, Mild allergic rhinitis; MSAR, moderate–severe allergic rhinitis; MS, mass spectrometry; NMR, nuclear magnetic resonance; NAE: N-acetylethanolamine; NR, Inhalant allergen-negative group; PAF: Platelet-activating; PE: Phosphatidylethanolamines; PS: Phosphatidylserine; VAS, visual analog scale; PR, Inhalant allergen-positive group; S1P, sphingosine-1-phosphate; TCA, Tricarboxylic acid cycle; TG: triacylglycerol; TNSS, total nasal symptom score.

**Table 3:** Metabolomes and metabolic pathways as diagnostic and severity markers for allergic rhinitis.

Metabolite	Diagnosis of AR		Severity of AR			Citation
	Pathway Analysis	ROC Analysis (AUC > 0.9)	Pathway Analysis	Correlation Analysis	ROC Analysis (AUC > 0.9)	
Lipid	Fatty acid Glycerophospholipid Sphingolipid		Fatty acid Sphingolipid		Increased S1P Decreased Linoleic acid	[10] ^1^
	Glycerophospholipid Linoleic acid Alpha- Linoleic acid Glycerolipid Arachidonic acid	Increased FA 30:7 Increased LPC(O) 18:1 Increased DAG 34:0 Increased LPC 18:0 Increased LPC 16:0		Increased DAG Increased LPC Increased TG Increased FA		[11] ^2^
	Linoleic acid Arachidonic acid					[13]
	Arachidonic acid					[14]
Amino acid and peptide	Arginine and proline				Increased Sarcosine	[10] ^1^
Nucleic acids	Pyrimidine				Increased Cytidine	[10] ^1^
	Purine					[14]
Others	Caffeine					[13]
	Porphyrin Chlorophyll					[14]
Combined multiple metabolites		Combined ^3^ Isovaleric acid Ethanol Acetylcarnitine Urine alanine Urine N,N-dimethylglycine, Urine chlophedianol				[17]

^1^ Severity of AR based on total nasal symptom score and visual analog scale; ^2^ Severity of AR based on serum IgE and IL-33 level; ^3^ AUC > 0.8; DAG: Diacylglycerol; FA: Fatty acid; LPC: Lysophosphatidylcholines; LPC O: Ether-linked lysophosphatidylcholine; TG: Triacylglycerol.

**Table 4:** Metabolome alterations in allergic rhinitis after allergen immunotherapy: Evidence from clinical studies.

Study Population	Sensitization	Allergen Immunotherapy	Method and Sample	Categories of Metabolites/Metabolic Pathways	Changes in Metabolites		Interpretation	Citation
					Increase	Decrease		
Ineffective group ^1^: 32.10 ± 14.77 (10) vs. Effective group ^2^: 27.30 ± 12.40 (33)	*Artemisia sieversiana* pollen	Allergen extracts: 0.5 mL of standardized *Artemisia sieversiana* allergen extracts (1.75 mg/5 mL) Route: SCIT Maintenance schedule: Twice a week for 1 year	Untargeted LC/MS and GC/MS in serum	Effective group vs. Ineffective group			Alterations in taurine- hypotaurine, pentose-glucuronate, pentose phosphate pathway, alanine, aspartate, and glutamate metabolism detected in serum were associated with SCIT, in which serum hypotaurine, taurine, and l-alanine levels were the robustest markers.	[66]
				Amino acids, peptides, and their related-metabolites		L-prolinamide Homocitrulline DL-norvaline Cysteinylglycine L-alanine L-norvaline L-threo-beta-hydroxy aspartic acid		
				Fatty acids and fatty acyls	Indole-3-acetic acid 3-hydroxypyruvate Palmitoleic acid Acar (18:2), (18:1)	5-aminovaleric acid Succinic acid semialdehyde ACar (8:1), (22:6), (12:1), (14:2), (14:1), (16:1), (20:4), (18:2), (16:0)		
				Glycolysis and sugars	Glyceric acid Xylitol Maltose	D-ribulose Gluconic acid Deoxy-galactose		
				Phospholipids	PI (36:4), (36:2), (38:4), (36:5e), (34:0) PC (16:0e), (18:0e), (20:5e), (18:2e), (20:4e), (17:0e) LPC (20:5), (18:2), (18:1), (18:0)	PI (36:3), (36:2) PE (18:0), (19:0), (20:0), (38:6), (40:8e), (36:4), (34:2), (36:5e), (34:3e), (36:2), (36:4e), (36:2), (40:6e), (36:2e), (36:3e) LPE (18:2), (18:1), (18:0) PC (18:0e), (16:1e) (18:2e), (16:0e), (18:1e) LPC (18:2), (18:0), (16:0)		
				Nucleic acids	Uridine, 2′-deoxyadenosine (3TMS)	Xanthosin Phenobarbital Cyclobarbital		
				TCA cycle	D-fructofuranose 6-phosphate Inositol-1-phosphate, myo-			
				Other organic acids	Taurine	Hypotaurine, L-3,4-dihydroxymandelic acid Ferulic acid cis		
				Others	D-glucarate Homogentisic acid Alpha-tocopherol	P-cymene Hydroxylamine Dopamine Pyrogallols 2-methylHippuric acid		
SM-SCIT: 11.00 (IQR 2.5) (63) vs. DM-SCIT: 10.50 (IQR 6.3) (62)	Der p and Der f	Allergen extracts ^3^: SM-SCIT Der p VS DM-SCIT: Der p:Der f = 1:1 Route: SCIT Maintenance schedule: 36–42 weeks	Targeted LC/MS in serum	Post SM-SCIT vs. Baseline			Alterations in omega-6-related arachidonic acid and linoleic acid metabolism detected in serum were associated with treatment response of SCIT.	[67]
				Arachidonic acid		15(S)-HETE 5(S)-HETE 12(S)-HEPE		
				Linoleic acid		9(S)-HPODE 13-HODE		
				Post DM-SCIT vs. Baseline				
				Arachidonic acid		15(S)-HETE 11(S)-HETE 8(S)-HETE 5(S)-HETE 12(S)-HEPE 5(S)-HEPE		
				Linoleic acid		13-HODE		
				Alpha-Linoleic acid		EPA Alpha-linolenic acid		
				Effective group vs. Ineffective group (VIP > 2)				
Ineffective group: 32.7 ± 8.3 (29) vs. Effective group ^4^: 30.30 ± 9.3 (39)	Der f ± Der p	Allergen extracts: 100 μL daily of standardized allergen Der f drops (1000 μg/mL) Route: SLIT drop Maintenance schedule: 3 years	Targeted LC/MS	Amino acids, peptides, and their related-metabolites	L-phenylalanine Nitrotyrosine	Ornithine ^5^ Glycine	The alterations in glycolysis, pyruvate-arginine-proline, and fatty acid metabolism detected in serum were associated with efficacy of SLIT.	[68]
				Fatty acids and fatty acyls	Linolenic acid ^5^	Arachidonic acid ^5^ Creatinine ^5^		
				Phospholipids		Sphingosine ^5^		
				Nucleic acids		Flucytosine Xanthine		
				Other organic acids		Lactic acid ^5^ Urea		
Placebo group: 36 ± 10 (14) vs. Active group: 36 ± 10 (8) Monosensitization (10) vs. Polysensitization (12)	Grass-pollen (*Phleum pratense*)	Allergen extracts: GRAZAX® (*Phleum pratense*, 75,000SQ-T tablets) Route: SLIT Maintenance schedule: 2 years	Untargeted LC/MS and GC/MS in serum	Active vs. Placebo T2			Lysophospholipid, bile acid, and fatty acid metabolism detected in serum were altered after SLIT.	[69]
				Amino acids, peptides, and their related-metabolites		Tyramine Phe-Phe		
				Fatty acids and fatty acyls	C 22:6 (DHA)	C 18:0 (Stearic Acid)		
				Phospholipids	LPC (18:2), (20:4)	LPC (12:0) PC (O-15:0/20:4)		
				Bile acids	Glycochenodeoxycholic Acid 3-glucuronide Taurocholic Acid 3-sulfate	Ursodeoxycholic Acid		
				Others	Thymol Sulfate	P-cresol Hydrogen sulfite		
				Poli vs. Mono T0				
				Amino acids, peptides, and their related-metabolites		Trp-Leu		
				Fatty acids and fatty acyls		Arachidonic Acid Adrenic Acid Linoleic Acid Linolenic Acid Tetradecanedioic Acid		
				Phospholipids		Lyso-PAF C:16 Lyso-PAF C:18 LPC (16:1) PC (18:1/13:0) PE (14:0/12:0) PS (24:1/22:4)		
				Other organic acids	Citric Acid			
				Bile acids	Glycochenodeoxy cholic acid 3-sulfate			
				Poli-Active vs. Mono-Active T2				
				Amino acids, peptides, and their related-metabolites	Trans-4-hydroxy-l-proline			
				Bile acids		Deoxycholic acid 3-glucuronide		
				Others	Benzoic Acid 3-keto-sphingosine	Levoglucosan Erythritol		
Ineffective group ^1^: 32.10 ± 14.77 (10) vs. Effective group ^2^: 27.30 ± 12.40 (33)	*Artemisia sieversiana*	Allergen extracts: standardized *Artemisia sieversiana* (1.75 mg/5mL) Route: SCIT Maintenance schedule: 1 year	Untargeted LC/MS and GC/MS in serum	Pre vs. post treatment			SCIT was associated with the alterations in amino acid, fatty acid, glycolysis, phospholipid, nucleic acid and organic acid metabolism detected in serum, in which serum L-tyrosine level was the most robust marker.	[70]
				(Effective group)				
				Amino acids, peptides, and their related-metabolites	DL-threo-b-hydroxyaspartic acid L-pyroglutamic acid L-tyrosine	L-valine		
				Fatty acids and fatty acyls	ACar(8:1), (22:6), (14:2), (20:4) Gamma-linolenic acid Succinic semialdehyde 5-aminovaleric acid			
				Glycolysis and sugars	D-ribulose			
				Phospholipids	PI (36:4), (34:1), (38:4), (36:2) PE (38:6), (38:7e), (36:4), (38:6e), (34:2), (36:5e), (40:8e), (36:4e), (38:4), (34:3e), (40:7e), (38:5e), (36:2), (38:5e), (36:3e), (36:2e) LPE (18:2), (18:0) LPC (16:0)	PC (18:2e), (20:4e)		
				Nucleic acids	3-methyladenine			
				Other organic acids	Hypotaurine Ferulic acid			
				Others	P-cymene 4-methylbenzoic acid			

^1^ Therapeutic index ≤25% admitted to the ineffective group.; ^2^ Therapeutic index ≥66% admitted to the effective group.; ^3^ No mention about the exact dose; ^4^ Patient obtained at least 30% reduction in symptom and medication score (SMS) compared to baseline; ^5^ Area under the curve (AUC) value of each metabolite should be higher than 0.7 for predicting AIT effectiveness; ACar, Acylcarnitines; AR, allergic rhinitis; CL, Cardiolipin; COX, Cyclooxygenase; Der f, Dermatophagoides farina; Der p, Dermatophagoides pteronyssinus; DAG, diacylglycerol; DHA, Docosahexaenoic acid; DM-SCIT, Double-mite subcutaneous immunotherapy; EPA, Eicosapentaenoic acid; FA, Fatty acids; GCDCA, Glycochenodeoxycholic Acid; GC/MS: Gas chromatography coupled with mass spectrometry; GPx, Glutathione peroxidase; HETE, Hydroxyeicosatetraenoic acid; HEPE, Hydroxyeicosapentaenoic acid; HHTrE, Hydroxyheptadecatrienoic acid; HPODE, Hydroperoxylinoleic acid; HODE, Hydroxyoctadeca- dienoic acid; HOTrE, Hydroxyoctadecatrienoic acid; LC/MS, Liquid chromatography coupled with mass spectrometry; Leu, Leucine; LOX, Lipoxygenase; LPI, Lysophosphatidylinositols; LPC, Lysophosphatidylcholines; LPC(O), Ether-linked lysophosphatidylcholine; LPE, Lysophosphatidylethanolamines; MS, Mass spectrometry; NAE, N-acetylethanolamine; OVA, Ovalbumin; PAF, Platelet-activating factor; PC, Phosphatidylcholine; PE, Phosphatidylethanolamines; Phe, phenylalanine; PI, Phosphatidylinositols; PS, Phosphatidylserine; SCFAs, Short-chain fatty acids; SM-SCIT, Single-mite subcutaneous immunotherapy; SMS, symptom and medication score; SPF, Specific pathogen-free; TCDCA, Taurochenodeoxycholate; Trp, Tryptophan; UCA, Urocanic acid; VIP, Variable importance plot; ROC, Receiver operating characteristic; RQLQ, Rhinitis conjunctivitis quality of life questionnaire; TG, Triacylglycerol.

**Table 5:** Metabolomes and metabolic pathways as allergen immunotherapy response markers for allergic rhinitis.

Metabolites	Effectiveness of Allergen Immunotherapy			Citation
	Pathway Analysis	Correlation/Univariate Analysis	ROC Analysis (AUC > 0.7) for Effective Group	
Lipids		Decreased 11-dehydroTXB2 Decreased 11(S)-HETE Decreased 13-HODE Decreased 15(S)-HETE Decreased 5(S)-HETE Decreased 8(S)-HETE		[67] ^2^
	Fatty acid		Decreased arachidonic acid Increased linolenic acid Decreased sphingosine	[68] ^3^
	Arachidonic acid			[69] ^4^
Amino acids, peptides, and their related- metabolites	Alanine, aspartate and glutamate Taurine and hypotaurine	Decreased hypotaurine Decreased L-alanine Increased taurine		[66] ^1^
	Arginine and proline		Decreased creatinine Decreased ornithine	[68] ^3^
	Alanine, aspartate and glutamate Aminoacyl tRNA Arginine and proline Butanoate Nitrogen Phenylalanine, tyrosine and tryptophan Taurine and hypotaurine			[70] ^1^
Glycolysis	Glycolysis		Decreased lactate	[68] ^3^
	Glycolysis			[69,70] ^1,4^
Pentose phosphate pathway	Pentose phosphate pathway			[66,70] ^1^

^1^ Efficacy of SCIT based on therapeutic index (≥66%); ^2^ Efficacy of SCIT based on rhinitis conjunctivitis quality of life questionnaire; ^3^ Efficacy of SCIT based on symptom and medication score (≥30% reduction); ^4^ Efficacy of SCIT based on reduction in mast cells and phagocytes; HETE: Hydroxyeicosatetraenoic acid; HODE: Hydroxyoctadecadienoic acid; TXB2: Thromboxane B2.

## Data Availability

No new data were created or analyzed in this study. Data sharing is not applicable to this article.

## Abbreviations

The following abbreviations are used in this manuscript:

12,13-EpOME	12,13-cis-Epoxyoctadecenoic acid
13(S)-HPODE	13(S)-Hydroperoxyoctadecadienoic acid
8S,15S-diHPETE	8S,15S-dihydroperoxy-5Z,9E,11Z,13E-eicosatetraenoic acid
9-HpETE	9-hydroperoxy-5,7E,11Z,14Z-eicosatetraenoic acid
9R,10S EpOME	(9R,10S)-9,10-Epoxyoctadecenoic acid
9,10 DiHOME	(Z)-9,10-Dihydroxyoctadec-12-enoic acid
∝	Association
AA	Arachidonic acid
ACar	Acylcarnitines
AIT	Allergen immunotherapy
AR	Allergic rhinitis
AS	Asthma
Ass	Association
AUC	Area under the curve
Bregs	Regulatory B cells
C	Control
Cer	Ceramide
CerP	Ceramide 1 phosphate
CL	Cardiolipin
COX	Cyclooxygenase
CTLA-4	Cytotoxic T-lymphocyte–associated protein
CTP	Cytidine triphosphate
DAG	Diacylglycerol
Der f	Dermatophagoides farinae
Der p	Dermatophagoides pteronyssinus
DG	Diacylglycerol
DGK	Diacylglycerol kinase
DHA	Docosahexaenoic acid
DHAP	Dihydroxyacetone phosphate
DM-SCIT	Double-mite subcutaneous immunotherapy
EPA	Eicosapentaenoic acid
FA	Fatty acid
GC	Gas chromatography
GC/MS	Gas chromatography coupled with mass spectrometry
GCDCA	Glycochenodeoxycholic acid
GPx	Glutathione peroxidase
H2S	Hydrogen sulfide
HDM	House dust mite
HEPE	Hydroxyeicosapentaenoic acid
HETE	Hydroxyeicosatetraenoic acid
HHTrE	Hydroxyheptadecatrienoic acid
HODE	Hydroxyoctadecadienoic acid
HOTrE	Hydroxyoctadecatrienoic acid
HPODE	Hydroperoxylinoleic acid
IgA	Immunoglobulin A
IgE	Immunoglobulin E
IgG	Immunoglobulin G
IL	Interleukin
ILC2s	Innate lymphoid cells
LC	Liquid chromatography
LC/MS	Liquid chromatography coupled with mass spectrometry
LC/MS/MS	Liquid chromatography coupled with tandem mass spectrometry
Leu	Leucine
LOX	Lipoxygenase
LPC	Lysophosphatidylcholines
LPC O	Ether-linked lysophosphatidylcholine
LPE	Lysophosphatidylethanolamines
LPI	Lysophosphatidylinositols
LPS	Lipopolysaccharides
LTD4	Leukotriene D4
LTE4	Leukotriene E4
MAR	Mild allergic rhinitis
MS	Mass spectrometry
MSAR	Moderate–severe allergic rhinitis
NAE	N-acetylethanolamine
NMR	Nuclear magnetic resonance spectroscopy
NR	Inhalant allergen-negative group
OVA	Ovalbumin
PAF	Platelet-activating factor
PC	Phosphatidylcholine
PE	Phosphatidylethanolamines
PG	Prostaglandins
PGD2	Prostaglandin D2
PGE	Prostaglandin E
PGE1	Prostaglandin E1
Phe	Phenylalanine
PI	Phosphatidylinositols
PR	Inhalant allergen-positive group
PS	Phosphatidylserine
PUFA	Polyunsaturated fatty acid
RQLQ	Rhinitis conjunctivitis quality of life questionnaire
ROC	Receiver operating characteristic
S1P	Sphingosine-1-phosphate
SCF	Short-chain fatty acids
SM-SCIT	Single-mite subcutaneous immunotherapy
SPF	Specific pathogen-free
TCA	Tricarboxylic acid
TCDCA	Taurochenodeoxycholate
TG	Triacylglycerol
TGF-β	Transforming growth factor-β
Th1	T-helper 1
Th17	T-helper 17
Th2	T-helper 2
Trp	Tryptophan
TSLP	Thymic stromal lymphopoietin
TSSS	Total nasal symptom score
TXB2	Thromboxane B2
UCA	Urocanic acid
VAS	Visual analog scale
VIP	Variable importance plot
